# Predation of young tortoises by ravens: the effect of habitat structure on tortoise detectability and abundance

**DOI:** 10.1038/s41598-020-58851-5

**Published:** 2020-02-05

**Authors:** Amalia Segura, José Jimenez, Pelayo Acevedo

**Affiliations:** 1 Instituto de Investigación en Recursos Cinegéticos, IREC (CSIC-UCLM-JCCM). Ronda de Toledo, 12, 13071 Ciudad Real, Spain; 2 Escuela Técnica Superior de Ingenieros Agrónomos (UCLM), Ronda de Calatrava, 7, 13071 Ciudad Real, Spain

**Keywords:** Biodiversity, Behavioural ecology

## Abstract

The predation of young tortoise is considered a major cause of mortality for many tortoise species. The predation by common ravens has been identified as being responsible for significant decreases in tortoise populations. Mediterranean spur-thighed tortoise hatchlings and juveniles in Maamora forest (Morocco) were studied in order to describe the size/age class predation of common ravens on young tortoises and infer the drivers of predation risk and population abundance. The results showed a high level of predation on young tortoises (<75 mm carapace length) attributed to ravens in areas with low vegetation cover, representing 100% of the cases of mortality (n = 147), but it was moderate in covered areas (n = 19), representing 12–27%. The population structure of living juveniles differed significantly between covered and uncovered areas, thus suggesting that raven predation might modify juvenile population structure. Finally, N-mixture models showed a positive relationship between (i) bare cover and tortoise detectability that is only evidenced when the plot is far from a perch and (ii) population abundance and shrub species-richness, being higher in uncovered areas. Our results improve the knowledge on predation and survival on this critical stage in life, which is crucial for the conservation of the Mediterranean spur-thighed tortoise.

## Introduction

Tortoise populations are characterised by high adult survival and low recruitment rates, which probably influence their demographic dynamics^1^. The lack of reliable information on recruitment complicates evaluations of the real importance of young individuals as regards population demography^2^. In this respect, threats that limit recruitment bouts may have an important regulatory effect on population dynamics^3^, especially when these threats continue over long periods of time (e.g.^4^).

Hatchlings and juveniles are more vulnerable than adults owing to their smaller size and higher susceptibility to environmental conditions, such as the temperature or rain, which affect their physiology, but also to vegetation cover, which influences thermoregulation, the availability of food and the risk of predation^3,5,6^. Indeed, the low temperature and high rains of winter^2,7^ and the high temperature of summer^8,9^ are considered some of the most common causes of mortality in hatchlings. The predation of young tortoises –their shell is soft or not sufficiently solid to protect them from predators– by mammals^7,10,11^ and birds^12–14^ is also considered a major cause of mortality in many tortoise species, which was particularly studied in North America^7,10,12,15,16^.

The common raven *Corvus corax* is considered to be one of the most relevant predators of tortoises and is responsible for 70–91% of the mortality of desert tortoises *Gopherus agassizii*^4,13,17^. Both single individuals and breeding pairs have been identified as responsible for significant decreases in tortoise populations, also in addition to modifying population structure by affecting juvenile size/age classes.^12,18,19^ Indeed, its high numbers and opportunistic feeding habits have severe impacts on its target prey, whose population size may be reduced without affecting the condition of the predator, which will switch to another prey when this resource becomes scarce (e.g.^4^). But the prey species has mechanisms by which to reduce predation. Vegetation cover has been documented as a key determinant as regards reducing young tortoises’ detectability, since it facilitates their camouflage^20^. Crypsis has anti-predatory benefits, and refuge areas, such as those areas dominated by vegetation cover, may reduce the risk of predation. It has also been documented that ravens’ predation on tortoises differs according to their spatial distribution of single individuals or breeding pairs. Tortoise predation is greater in adjacent human developments, which attract large numbers of single ravens, and in adjacent successful nests of breeding pairs throughout human developed and undeveloped areas^4^. Even the success of juvenile tortoise releases is compromised in the head-starting programs included in conservation strategies owing to the fact that certain ravens are attached to the predation of certain tortoise size classes^17,21^. It could, therefore, be hypothesized that the risk of raven predation on young tortoises might be high in areas with high dense tortoise populations and mediated by the overlaid effect of raven presence and vegetation cover.

Maamora forest, an anthropogenic cork oak forest located in northern Morocco, is considered to be close to the optimum niche –the core range– of the Mediterranean spur-thighed tortoise’s distribution *Testudo graeca*^22^ and one of the areas with the highest density populations documented to date^23^. However, the common raven has, over the last 40 years, increased its numbers and distribution worldwide, and this cork oak forest is no exception^24^. This increase in predators may be a threat to the Mediterranean spur-thighed tortoise, and especially to highly susceptible young individuals. Concretely densities of 0.8 breeding pairs km^−2^ had been observed in the lustrum (A. Segura unpublished data). In this context, our specific objectives were to: (i) describe predation and size classes’ preference of common ravens on young tortoises, and (ii) infer the main drivers of predation risk and abundance. These goals are relevant as regards improving knowledge on the predation and survival of the Mediterranean spur-thighed tortoise at this critical stage in its life and, therefore, the conservation of this threatened species.

## Results

### Raven predation on hatchlings and juveniles

Twenty-two occurrences of ravens, not including groups (7, 3, 6 and 6, in A, B, C and D, respectively), were detected on our four study sites (Table 1). Three raven nests with active breeding pairs were found and two breeding pairs had between 1 and 2 chicks (Table 1).

**Table 1:** Bare ground cover (%), shrub cover (%), shrub height (cm) and shrub species richness; raven perch and nest distance (m) in the four study sites, A, B, C and D (mean ± SD); maximum raven occurrence in the Mediterranean spur-thighed tortoise survey distributed by single individuals, breeding pairs and groups of >2 individuals (number of individuals); live (<100 mm carapace length, CL), dead (<100 mm CL) and raven predated tortoises (<75 mm CL) (in all of them, the number of individuals).

	A	B	C	D
*Vegetation*				
Bare ground cover	0	0 ± 0.2	17.9 ± 17.4	21.4 ± 13.8
Shrub cover	59.7 ± 21.1	52.0 ± 14.8	45.0 ± 23.0	28.2 ± 26.1
Shrub height	62.1 ± 17.7	71.1 ± 30.6	31.9 ± 27.3	19.8 ± 17.6
Shrub richness	2.0 ± 0.8	1.5 ± 0.7	0.8 ± 0.4	0.7 ± 0.7
*Predation risk*				
Perch distance	203.6 ± 35.4	510.8 ± 120.8	72.5 ± 36.3	89.2 ± 35.3
Nest distance	999.0 ± 84.5	1076.7 ± 131.1	184.9 ± 89.2	144.7 ± 37.7
*Raven occurrence*				
Single individuals	5	2	4	5
Breeding pairs	2	1	2	1
Groups	1(28)	1(5)	0	1(17)
*Number of live tortoises*	39	30	65	39
*Number of dead tortoises*	11	8	55	92
*Raven predated tortoises*	3	1	55	92

**Figure 1:**
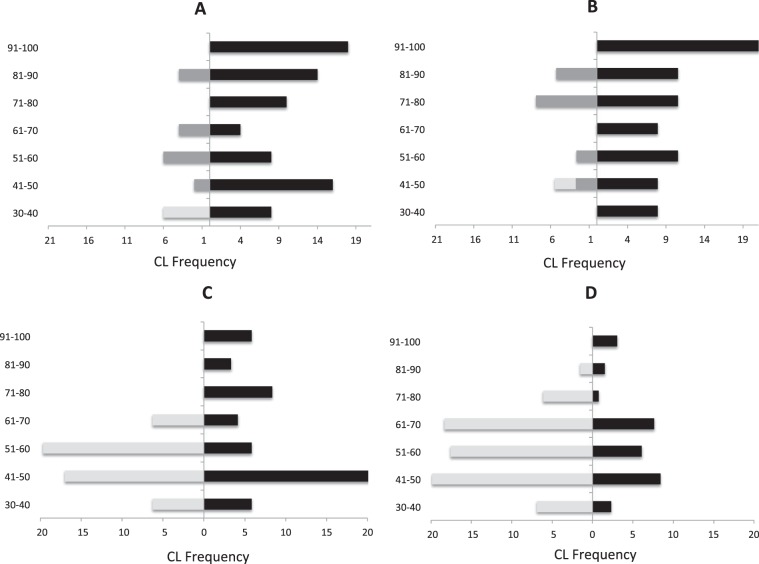
Distribution of carapace length (mm; CL) in the four study areas: covered (**A,B**) and uncovered (**C,D**). Data represent the frequencies of young tortoises: live shown in black, dead by raven predation in light grey and dead by any other reason in dark grey.

One hundred and sixty-six dead young tortoises (<100 mm Carapace Length; hereafter CL) (11, 8, 55 and 92, in A, B, C and D, respectively) were detected during the surveys. Most of the dead juvenile tortoises found (88%) were located in the uncovered areas (areas C and D), where the main CL size category of dead tortoises corresponded to between 41 and 70 mm (Fig. 1). The cause of mortality in those areas was associated with predation by the common raven (100%, n = 147), where only < 75 mm CL tortoise carcasses were found with signs of raven predation under perch and nest trees. The ravens ate the hatchling and juvenile tortoises by pulling off their head and limbs (6%) or pecking holes through the carapace (60%) or plastron (34%). Indeed, 74 and 15 juvenile tortoises were predated by two active breeding pairs of ravens in spring 2018 (in areas D and C, respectively). Nevertheless, unknown causes of mortality dominated in the covered areas, and only 12–27% were related to raven predation. The threshold size above which young tortoises are safe from raven predation in both covered and uncovered areas was 75 mm CL. Dead young tortoises associated with raven predation differed significantly between covered and uncovered areas (A *vs*. C: X^2^ = 4.07, p < 0.05, n = 66; A *vs*. D: X^2^ = 4.25, p < 0.05, n = 103; B *vs*. C: X^2^ = 5.05, p < 0.05, n = 63; B *vs*. D: X^2^ = 5.19, p < 0.05, n = 100), but did not differ between either covered (A *vs*. B: X^2^ = 0.08, p = 0.77, n = 19) or uncovered areas (C *vs*. D: X^2^ = 0, p > 0.99, n = 147).

### Live hatchlings and juveniles

One hundred and sixty-three live young tortoises (39, 30, 65, 39 in A, B, C and D, respectively) were found during the surveys. Figure 1 shows the size-class distribution on the four study sites. The juvenile structure of live tortoises did not differ between the covered areas (A *vs*. B; X^2^ = 7.68, p = 0.26, n = 69) but differed significantly between the uncovered areas (C *vs*. D; X^2^ = 21.19, p < 0.05, n = 104) and also between the covered and the uncovered areas (C *vs*. A: X^2^ = 16.06, p < 0.05, n = 104; C *vs*. B: X^2^ = 16.06, p < 0.05, n = 95; D *vs*. A: X^2^ = 36.89, p < 0.05, n = 78; D *vs*. B: X^2^ = 36.77, p < 0.05, n = 69). The main differences concerned the CL size category of 81–100 mm, which represented 41% versus 16% in the covered and uncovered areas, respectively.

### Tortoise detectability and abundance: risk of predation by ravens

According to previous results regarding dead animals found on our study sites, only those animals < 75 mm CL were susceptible to predation in Maamora forest. Predation risk analysis was, therefore, restricted to these size classes: 140 detections (A: 27 tortoises in 11 occupied grids; B: 21 tortoises in 10 occupied grids; C: 57 tortoises in 13 occupied grids, and D: 35 tortoises in 7 occupied grids). It varied from a maximum of 3 to 5–11 tortoises per grid in covered and uncovered areas, respectively (Table 1).

In the N-mixture model, we used a negative binomial model (Table 2). The stepwise procedure carried out to select predictors explaining detectability and abundance processes is summarized in Table 2. The bootstrap *p*-values for the final model based on the SSE, Freeman-Tukey, and Chi-square statistics were 0.03, 0.00 and 0.01, respectively. The value of *ĉ* (ratio of observed/expected) was 1.59.

**Table 2:** Model selection of Mediterranean spur-thighed tortoise hatchlings and juveniles (<75 mm carapace length; see text for details): (1) Latent abundance distribution and (2) covariates of abundance and detection. Covariates considered: shrub richness; site, bare ground cover (%) and raven perch distance (m). Model selection based on Akaike’s Information Criterion (AIC), number of parameters (nPars), the difference in AICc from the best fitted models (ΔAICc < 2), model weights (AICwt), and cumulative model weights (cltvWt).

Model specification	nPars	AIC	ΔAICc < 2	AICwt	cltvWt
*(1) Latent abundance distribution*					
Negative binomial	3	541.51	0.00	1.00	1.00
Zero Inflated	3	584.01	42.50	0.00	1.00
Poisson	2	620.03	78.52	0.00	1.00
*(2) Covariates of abundance (γ) and detection (p)*					
*γ* (Shrub richness + Site) *p* (Bare ground cover*Perch distance)	10	527.82	0.00	0.98	0.98
*γ* (Site) *p* (Bare ground cover*Perch distance)	9	536.24	8.42	0.01	0.99
*γ* (.) *p* (Bare ground cover*Perch distance)	6	538.68	10.86	0.00	1.00
*γ* (.) *p* (.)	3	541.51	13.69	0.00	1.00

**Figure 2:**
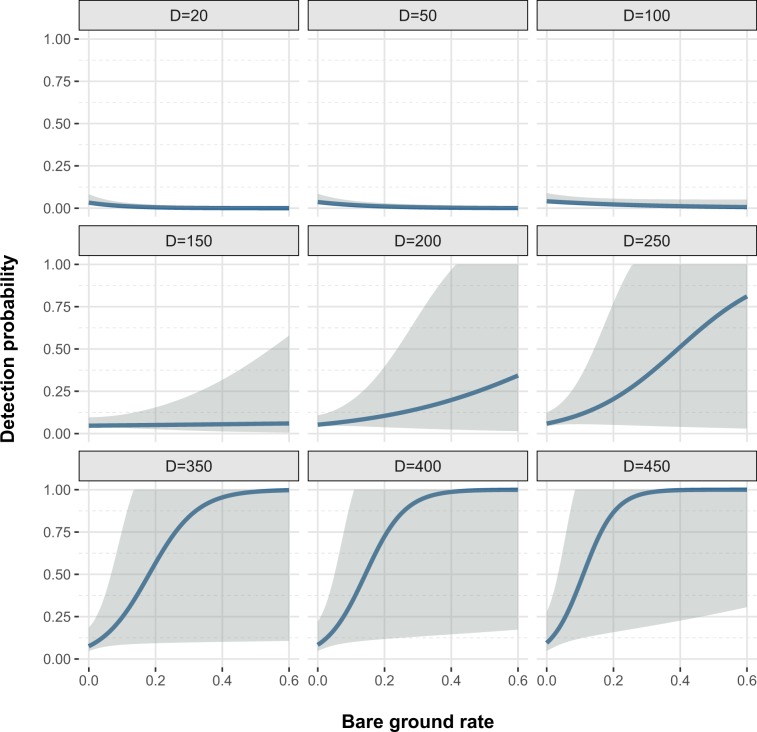
Interaction between two continuous covariates: bare ground cover rate and distance to the nearest raven perch (D, in meters) over the probability of detection.

**Figure 3:**
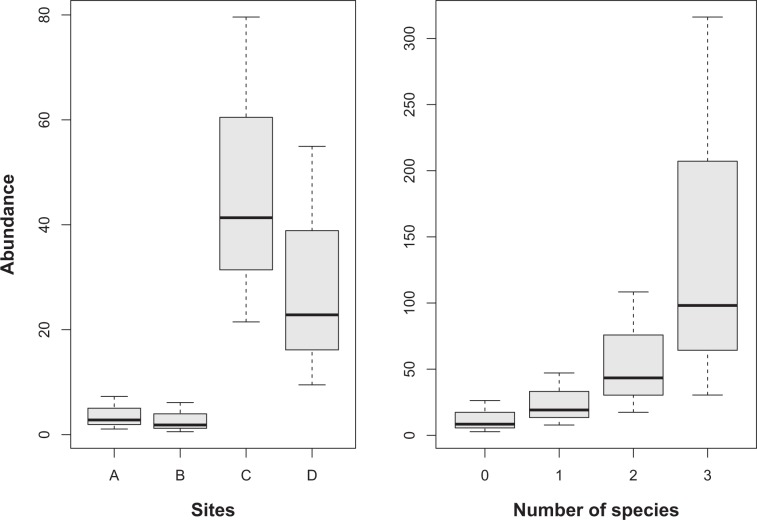
Relationship between sampling sites (A, B, C and D; left graph) and the richness of shrub species (number of species; right graph) with the abundance of Mediterranean spur-thighed tortoise hatchlings and juveniles (<75 mm carapace length).

The final model included the interaction between bare ground cover and the distance to the raven perch in the detection process (Fig. 2), and the site and the number of shrub species in the abundance process (Fig. 3, Table 3). The detectability increased with the percentage of bare ground, but this effect was apparent only when the distance to a raven perch was greater. The abundance was higher in uncovered areas and in grids with high shrub species richness.

**Table 3:** Summary and statistical parameters of the final N-mixture model parameterized to estimate Mediterranean spur-thighed tortoise hatchlings and juveniles (<75 mm carapace length) as regards both detectability and abundance (significance codes: ns no significative; ** < 0.01 and *** < 0.001).

Process	Variable	Estimate	Standard Error	Z	P (>|Z|)
Abundance	Intercept	1.025	0.49	2.094	***
	Shrub richness	0.709	0.222	3.196	**
	Site B	−0.419	0.619	−0.677	ns
	Site C	2.697	0.564	4.78	***
	Site D	2.102	0.586	3.588	***
Detection	Intercept	−2.416	0.454	−5.32	***
	Bare ground cover	0.636	0.516	1.23	ns
	Perch distance	1.9	0.588	3.23	**
	Bare ground cover* Perch distance	1.961	0.78	2.52	**

## Discussion

### Evidence of common raven predation on hatchlings and juveniles

This study reveals the predation of common ravens on juveniles of Mediterranean spur-thighed tortoises in certain areas of Maamora forest. Both, single individuals and breeding ravens were observed killing, carrying away and consuming juvenile tortoises, their preference being for class sizes 40–70 mm CL. The selection by ravens of certain size classes of tortoises over others has been found in other Testudinidae populations too^18,19^. Despite some tortoises being paint-marked to identify them as recaptures, no evidence of any increased risk of predation was observed for the recognisable fraction of the population (but see^25^); only in C uncovered area was one individual found dead, representing 2% of the marked individuals.

Bearing in mind that raven predation might vary between years and among individuals, and the fact that our study comprised only one year, our results still suggest that the predation of juvenile tortoise in the study area was higher when compared with that of populations of Mediterranean spur-thighed tortoises in southern Spain^8^. Indeed in those areas raven predation did not affect tortoise populations at all. Nevertheless, some similarities with other Testudinidae populations affected by raven predation were found^12,16,26^, although the ravens involved in predation in our study appeared to have a slightly lower size threshold above which juveniles are safe from predation (75 mm CL) than reported for other Testudinidae populations (85 and 100 mm CL; 27, 21 respectively). This might be associated with the length of time shell hardening takes in Mediterranean spur-thighed tortoises, which has been documented to limit the probability of predation by ravens (e.g.^27^), and this merits further studies.

Nevertheless, when comparisons of raven predation on tortoises were restricted to covered versus uncovered areas, in the former such occurrences were all anecdotal while high mortality rates were rigorously recorded in the latter. Certainly, we were surprised by the high amount of predation by one pair of breeding ravens, which predated 74 tortoises of <75 mm CL in a single breeding season. Indeed, raven predation might be modifying juvenile tortoise population structure through altering recruitment (e.g.^4^). Further studies are required to disentangle the role played by predation in tortoise population structure within the Maamora forest.

### Predation risk by ravens, population size and structure of young tortoises

In this study, tortoise detectability –associated with predation risk– was mediated by the interaction between predator presence and bare or low cover areas; with tortoise detectability increasing with greater areas of bare ground, mainly in locations far from perch trees (e.g.^18,28^). This might suggest that ravens could be modulating the behavioral response of young tortoises, e.g. they will reduce their activity in areas near perch trees in order to be less detectable by the ravens (e.g.^29^). In this respect, it is reasonable to assume that the survivorship of juveniles in areas with higher predation risk might be lower and, therefore, a lower juvenile population size will characterise populations in such areas. Conversely, we found a higher abundance of young tortoises in uncovered areas, where they suffered higher predation, but also in areas where there was high diversity of shrub species in both covered and uncovered areas (e.g.^30^). It is thus plausible to assume that many other factors involving juvenile physiological costs (e.g.^29^) or even female reproduction traits –the number of clutches, clutch size and recruitment success–^3^, might explain part of the variation found in the size of juvenile populations in covered and uncovered areas^3^. However, in higher risk predation areas, it might also be expected that juvenile population structure would be modified and show differences in size/age classes^12,18,19^. Indeed, the higher percentage of longer/older juveniles (76–100 mm) –which are not considered susceptible to predation– found in covered areas compared to in uncovered ones evidenced a threat to juvenile survivorship in the lower size/age classes in those areas where hatchlings are more detectable, e.g. uncovered areas, and whose effects, among others, might be mediated by vegetation cover (e.g.^30^).

Finally, hatchlings are challenging to study since they are rarely encountered in the field^31,32^ and, as such, very low capture rates and practically nonexistent recapture rates of Mediterranean spur-thighed tortoise hatchlings have been reported in southern Spain^33,34^. Further studies are required to discover the role played by predation in the recruitment process, which will have later impacts on the size and structure of tortoise populations. This will also help to disentangling the possible physiological costs associated with predation risk.

## Material and Methods

### Study area

The study was conducted in areas of low elevation (72–185 m a.s.l.) and sandy soil in Maamora forest (northwest Morocco; 34° 02′ 54.19″ N, 6° 27′ 19.24″ W). The climate is Mediterranean, with hot and dry summers, and the annual range of average rainfall is from 300 to 500 mm. Maamora forest is dominated by cork oak trees, *Quercus suber*, with scattered endemic wild pear, *Pyrus mamorensis*, wild olive *Olea europaea*, green olive *Phyllirea latifolia* and mastic *Pistacia lentiscus*, and a sparse understory represented by bush and shrub species, such as Mediterranean broom *Genista linifolia, Cytisus arboreus, Stauracanthus genistoides*, dwarf palm *Chamaerops humilis*, French lavender *Lavandula stoechas*, sage-leaved rockrose *Cistus salviifolius, Halimium halimifolium* and *Thymelaea lythroides*^35^.

**Figure 4:**
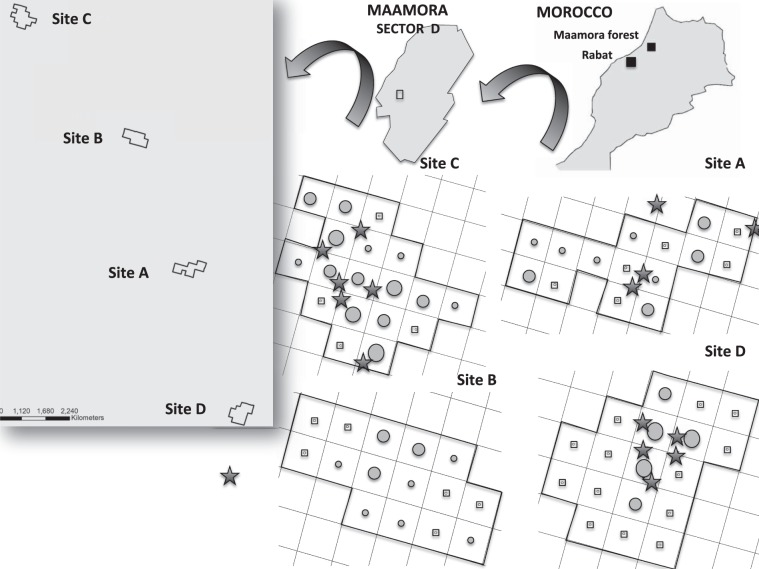
Location of the study area in Morocco and tortoise populations studied (sites A, B, C and D) is shown. Grey circles size is proportional to the maximum abundance of live young tortoises (<75 mm CL) observed per 1ha-grid (1, 2–3, 4–5, 6–11 ranges). Small grey squares mark grids where no tortoises were found. Black stars represent the raven perch or nest locations.

The study specifically took place on four sites that were close together (separated by 3 km), but always ensuring that the tortoise populations’ territories were separated by a sufficient distance for them to be considered as independent populations^34,36^; Fig. 4. Despite the fact that all the sampling sites were located on private land on which there has been no pet trade (>10 years protected) and that the undergrowth is well represented when compared with other sites in unprotected Maamora, the study sites differed as regards their vegetation cover. Two of them were characterized by high cover and the diversity of their shrub and herbs (hereafter denominated as covered areas, A and B), while the other two were characterized by a lower cover and diversity of shrubs and a high percentage of bare ground (hereafter denominated as uncovered areas, C and D; see Table 1 for further details). This experimental design allowed us to test for differences in tortoise detectability, and, therefore, infer predation risk, in relation to vegetation cover.

### Mediterranean spur-thighed tortoise and common ravens

The study sites were surveyed during the 2018-breeding period, comprising the end of February until the end of May, covering an area of 15 ha in each of the covered areas and of 18 ha in each of the uncovered ones. Each of the four sites was intensively surveyed in order to detect young tortoises. This was done for four (uncovered areas) or five (covered areas) days by four trained people. The survey consisted of searching the entire territory for the occurrence of individual tortoises, but focusing on the detection of hatchlings and juveniles (<100 mm CL). The tortoises were recorded from 12 h until 16 h on foot and in adequate weather conditions (sunny days with temperatures of between 20 and 24 °C). Each recorded individual was georeferenced using a GPS and the CL was measured using a vernier calliper (accuracy ± 1 mm). Size classes were used to characterize the tortoises’ population structure in covered and uncovered areas^24^. The Chi-square test was used to assess any differences between covered and uncovered areas in terms of hatchling and juvenile size-classes structure, considering measurements of 30–40, 41–50, 51–60, 61–70, 71–80, 81–90 and 91–100 mm. The tortoises’ carapaces were lightly marked with non-toxic paint so as to be able to identify any recaptured individuals. In addition, two additional days per area at the beginning and the end of the raven breeding season were carried out looking for any dead animals, which were collected and measured and, when possible, their cause of death was determined on the basis of a visual examination of the remains of the animal and the area in which it was found. These data were then used to determine the relevance of predation by ravens and to characterize the size-classes of the tortoises that are predated by this species.

During the tortoise survey, the location of common raven perches and nests were registered with a GPS. For this purpose, we also surveyed a buffer area of 1.5 km around the sampling areas, which is slightly greater than the average distance between the ravens’ territories^4^. The number of single individuals and breeding pairs of ravens was also recorded, along with their breeding status and their recruitment success (chicks or fledglings).

### Modelling tortoise detectability and abundance

The recorded data were referred to 1-ha grids, which were our territorial unit for analytical purposes. The number of young tortoises susceptible to predation (according to our data < 70 mm CL, see below; but see^21,37^) was, therefore, quantified (our response variable) for each grid and survey. In addition, each grid was characterized during the survey in relation to vegetation cover, and specifically to the shrub cover (%), shrub height (cm) and species richness, and the bare ground cover (%). In order to incorporate the effect of the common raven into species detectability, the mean distance to the nearest raven perch and nest (m; two variables) in each grid was also calculated.

We used N-mixture models to model the detectability and abundance of the hatchlings and juveniles that were, according to the count data, susceptible to predation, while we accounted for imperfect detection^38^ using the unmarked package^39^ in R^40^. Our assumption is that the detectability of young tortoises during the surveys can be considered a proxy of young individuals’ detectability by the ravens. Hatchlings and juveniles are well camouflaged and difficult to follow in their environment. Their detectability is very low^8,10,16,41^, since it is linked to the central hours of the day when animals are active. We, therefore, assume that detectability by observers is a proxy of detectability by ravens in order to be able to explore the effect of different factors on predation risk. This does not mean that the observer is able to detect the same number of young tortoises, but that the higher the detectability for the observer, the higher detectability for the ravens.

We used Akaike’s Information Criterion corrected for small sample sizes (AICc)^41^ to select the most appropriate error distribution by comparing the performance of Poisson, zero-inflated Poisson and Negative Binomial models. The detection and abundance processes were then modelled sequentially. A forward stepwise procedure was used to identify the most relevant predictors explaining the detection process. The same procedure was subsequently followed in order to identify the predictors explaining the abundance process. AICc was also used to compare models in the stepwise procedures by following the ΔAICc < 2 rules^42^. N-mixture models require an integer value that specifies the upper bound used in the integration (K). In our study, this upper bound was set at K = 120, which is sufficiently large for it not to have an effect on the model results. We used the parametric bootstrap approach (1000 samples) in unmarked to attain p-values from the sums of squares, along with Chi-square and Freeman-Tukey fit statistics as a measure of the goodness of fit of the final model.

### Ethic statements

Sampling of Mediterranean spur-thighed tortoises in Maamora forest was conducted under the authorization and following the protocols approved by Le Haut-Commissariat aux Eaux et Forêts et à la Lutte Contre la Désertification of Morocco (High Commission for Waters and Forests and the Fight against Desertification). The field surveys were done in accordance with the guidelines and regulations. There was non-invasive sampling.

## Data Availability

The datasets generated during and/or analyzed during the current study are available from the corresponding author on reasonable request.
